# Towards China-initiated actions on AI safety and governance

**DOI:** 10.1093/nsr/nwag204

**Published:** 2026-04-08

**Authors:** Yi Zeng, Tiejun Huang, Yugang Jiang, Mu-ming Poo

**Affiliations:** Beijing Institute of AI Safety and Governances, China; Beijing Key Laboratory of Safe AI and Superalignment, Institute of Automation, Chinese Academy of Sciences, China; Beijing Academy of Artificial Intelligence, China; State Key Laboratory of Multimedia Information Processing, Peking University, China; Fudan University, China; CAS Center for Excellence for Brain Science and Intelligence Technology, China

The rapid progress of Artificial Intelligence (AI) poses critical challenges to all sectors of society. It is well recognized that AI could help to accelerate the advancement of many fields, from science and technology to global economy, social welfare, and ecology. Yet there are also looming threats of AI development to the harmony and safety of all societies. It is particularly alarming that many pioneering AI labs and tech companies are racing to achieve Artificial General Intelligence (AGI) and superintelligence that could outperform humans in all tasks that people are capable of doing, without seriously considering how to prevent potential perils of this development. This calls for collective global deliberations on safe and human-aligned AI and effective actions for AI governance.

The geopolitical competition among AI superpowers, particularly between the United States and China, complicates the issue of AI governance. While Silicon Valley AI tech giants are racing towards AGI or superintelligence with unprecedented amounts of capital investment and technical prowess, Chinese AI companies and research labs are mainly focusing on broad commercial and scientific/technological applications. This apparent dichotomy in AI development is superficial, since AGI will inevitably invade all arenas of AI applications when it becomes available. By all accounts, this will very likely happen within the next 5 to 10 years. It is often argued that geopolitical competition and the commercial interests of tech giants will prevent meaningful dialogues and consensual global AI governance, since each competitor aims to win the race and has little trust in the other’s adherence to agreed guardrails and regulations.

During the cold-war period, efforts to deal with the pressing threat of nuclear war were initiated by concerned citizens and scientists from the United States and Soviet Union. Pugwash conferences, for example, were instrumental in opening communication channels during a time of strained US–Soviet relations, and they provided the ground work for a number of nuclear weapon-related treaties between the two countries. Since the emergence of large language models (LLMs), many efforts have been made on global AI safety and governance issues from both science and policy perspectives. The UN Global Dialogue on AI Governance and the Independent Scientific Panel on AI had contributed to the agenda of the United Nations General Assembly. Two international scientific reports on AI safety have been released, based on discussions led by Yoshua Bengio and participated in by >100 AI researchers. The World AI Conference and High-Level Meeting on Global AI Governance series provided an important platform for the exchange of ideas. The International Research Network on AI Development and Governance (AIR-Net), a scientist-driven collaborative network on development and governance of AI, was established. AI pioneers Geoffrey Hinton, Joshua Bengio, Stuart Russell and others have recently made an open statement, calling for a prohibition on the development of superintelligence, not lifted before there is broad scientific consensus that it will be done safely and controllably. This call has received strong international support, with co-signatures from more than 100 000 individuals from all sectors of society around the globe, including many Chinese professionals.

Despite all these efforts, no substantial step has been taken at the international level to develop effective and action-oriented AI governance. This could be attributed to the predominant commercial interests of AI companies and the lure of being the first to reach the AGI goal. Warnings of potential catastrophes and even existential risks abound, yet little actual progress has been made in instituting global guardrails and governance mechanisms. Two apparent difficulties remain: first, the lack of trust among competing big AI technology giants and between the US and China in each party’s commitment in adhering to global AI regulations; second, the absence of enforcement mechanisms for implementing guardrails and regulations once global consensus has been achieved.

We suggest that a realistic next step is for the Chinese government and AI community to take concrete actions in addition to and beyond mere global deliberations on principles and policies. In response to the UN COP climate change conferences, where extensive discussions at the policy level have been ongoing for nearly three decades, China has taken the initiative to implement step-by-step actions towards the goal for carbon peaking and neutrality. This involves government-initiated projects and regulations in developing renewable and green energy and has resulted in a steady progress in reducing carbon emission in the absence of corresponding efforts in Western advanced economies. We are confident that China could also take the lead to develop AI safety guardrails and implement effective governance mechanisms. This will demonstrate to the world, particularly geopolitical and economic rivals, that effective actions could be taken and that China’s commitment to AI safety is genuine and trustworthy. Like green energy technologies, China’s experience on AI safety and governance from technical and practical perspectives will be shared with the international community.

The actions to be taken are well-recognized, and efforts from the Chinese AI community could be: (1) to develop technical safety and security guardrails that prevent misbehaviors of AI systems and misuses by rogue AI users; (2) to develop technologies that instill human-aligned behaviors into large AI models and physical systems with embodied intelligence; (3) to develop relief technologies, emergency measures, and symbiotic social systems for addressing AGI; and (4) to openly share AI safety and security technologies with the global AI community and public as soon as they are developed. Scientists, AI developers, and concerned scholars should continuously support the Chinese government in several actions: (1) to strengthen existing, and establish new, AI safety expert committees that could closely monitor emerging AI technologies requiring safety guardrails/regulations and regularly provide action-oriented recommendations at all levels of government; (2) to regularly announce national AI safety guidelines and regulations based on recommendations of national AI safety expert committees; and (3) to install legal mechanisms that enforce the implementation of AI safety and governance regulations at the national level.

Initial actions have already been taken by the Chinese government, including: (1) the Interim Measures for the Management of Generative AI Services (effective since 2023), jointly issued by the Cyberspace Administration of China (CAC), National Development and Reform Commission (NDRC), Ministry of Education (MOE), Ministry of Science and Technology (MOST), Ministry of Industry and Information Technology (MIIT), Ministry of Public Security (MPS), and National Radio and Television Administration (NRTA); (2) the Measures for the Identification of AI Generated and Synthesized Content (effective since 2025), jointly issued by CAC, MIIT, MPS, and NRTA; (3) the AI Security Governance Framework (Version 2.0) released by National Technical Committee 260 on Cybersecurity of Standardization Administration of China (SAC), and National Computer Network Emergency Response Technical Team/Coordination Center of China (CNCERT); and (4) the AI safety and governance-related standardization released by the AI Standardization Technical Committee of MIIT (MIIT/TC1), and the National AI Standardization Expert Working Group. However, due to the rapid advance of AI technologies, timely amendments of AI safety regulations and governance mechanisms are highly challenging. A case in point is the recent widespread development and application of OpenClaw-like AI agents. In the absence of effective safety regulations and governance in place, adverse consequences are already emerging in society at large.

Sustainable development of AI technology needs societal concurrence that AI products are safe and aligned with human needs. In comparison with the rapid pace of development in frontier AI technologies and their applications, the progress of AI governance is alarmingly slow. That AI safety could depend solely on the self-control of AI developers is an illusion, given the well-recognized commercial or individual interests in the AI field. The argument that it will take disastrous events like a nuclear power plant accident before AI governance could be initiated is unacceptable. What we really need are the consistency, will, and discipline of governments and AI communities to take action in AI safety and governance. This is becoming the most critical issue in the frontier of science and technology. We here call for support for action-oriented AI governance by the Chinese government as well as the AI community in China and around the world, for achieving a prosperous and safe future for AI.

